# Adherence to Medical Treatment and Its Determinants Among Adult Saudi Glaucoma Patients in Riyadh City

**DOI:** 10.7759/cureus.6847

**Published:** 2020-02-02

**Authors:** Asem Shadid, Waleed Alrashed, Abdulelah Bin Shihah, Abdulrahman Alhomoud, Mushref Alghamdi, Abdulaziz Alturki, Abdulaziz Shadid, Essam Osman, Alanoud Alfaris, Rajiv Khandekar

**Affiliations:** 1 Medicine, Imam Mohammad Ibn Saud Islamic University, Riyadh, SAU; 2 Ophthalmology, Imam Mohammad Ibn Saud Islamic University, Riyadh, SAU; 3 Family Medicine, King Faisal Specialist Hospital and Research Center, Riyadh, SAU; 4 Ophthalmology, King Khalid Eye Specialist Hospital, Riyadh, SAU; 5 Internal Medicine, King Khalid University Hospital, Riyadh, SAU; 6 Medicine, King Saud University, Riyadh, SAU; 7 Ophthalmology, King Abdulaziz University Hospital, King Saud University, Riyadh, SAU; 8 Epidemiology and Public Health, Imam Mohammad Ibn Saud Islamic University, Riyadh, SAU; 9 Epidemiology and Public Health, King Khalid Eye Specialist Hospital, Riyadh, SAU

**Keywords:** adult saudi glaucoma

## Abstract

Background

Glaucoma in adults is a leading eye disease that causes blindness. Its management is life long and both surgical and medical treatment options are available to manage this ailment. Patients and their caregiver’s cooperation in instilling timely eye drops is crucial for the effective reduction of intraocular pressure (IOP) and by halting/delaying the progression of visual disabilities due to glaucoma. Periodic assessment and strict adherence to medical treatment has been found to be less than desired. It is influenced by the severity of the disease, the duration of the ailment, the number of eye drops being used, the literacy level, and the cost of medication. We present the adherence rate of topical medications and its determinants among adult Saudi glaucoma patients getting treated in 2017 in a tertiary eye center in Saudi Arabia.

Methodology

This cross-sectional survey was carried out from January to March 2017 at a tertiary eye hospital in Riyadh, Saudi Arabia. To undertake a cross-sectional study, we assumed that among 1300 patients visiting the eye department of a tertiary eye hospital, the level of non-adherence to glaucoma medication would be in 27% of glaucoma patients. To achieve a 95% confidence interval and an acceptable error margin of 5% for a survey, we needed to interview at least 253 participants.

Results

We interviewed 263 randomly selected glaucoma patients among 1236 patients visiting the eye department of the tertiary eye hospital in Riyadh, Saudi Arabia. Two-thirds of participants were school graduates, Saudi nationals, and had undergone surgery for glaucoma in the past; perhaps only YAG laser peripheral iridotomy (PI). Less than half of the participants (43%) had less than a one-year duration of glaucoma. The client-perceived subjective adherence rate to glaucoma medication was noted in 191/263 = 72.6% (95% Confidence Interval 67.2 -78.0). Of the 263 glaucoma patients, 229 judiciously abided with their follow-up appointments with ophthalmologists. Thus, the compliance to follow-up for glaucoma treatment was 87.1% (95% CI 83.0 - 91.1).

Conclusion

Our study with a large sample is perhaps the first one to assess compliance with medical treatment among adult Saudi glaucoma patients. The adherence rate for topical glaucoma medication measured using the subjective method was 72% among adult glaucoma patients. Adherence with the follow-up appointment with the glaucoma specialist was as high as 87%. Adherence with medical treatment found in the present study versus the literature review suggested that despite different sets of barriers, the adherence rate in Saudi adult glaucoma patients noted in our study was high. Knowledge, beliefs, and attitude are known to affect the adherence rate. In our study, education level and relatives having glaucoma were not associated with the adherence rate. This indirectly suggests that knowledge about the use of medication in the treatment of glaucoma that is gained by patients had a limited influence on adherence in our glaucoma patients. Modes of dispersing knowledge to elderly glaucoma patients and their impact on the adherence rates of medications for glaucoma management need to be further studied.

## Introduction

Glaucoma in adults is a leading blinding eye disease [1]. Its management is life-long and both surgical and medical treatment options are available to manage this ailment [2]. However, both ophthalmologists and glaucoma patients prefer medical management. Safe and less frequent dosage of topical glaucoma medications are the main reason for this preference [3]. Patients and their caregiver’s cooperation in instilling timely eye drops and the spacing between the drops are crucial for the effective reduction of intraocular pressure (IOP) and by halting/delaying the progression of visual disabilities due to glaucoma. Periodic assessment and strict adherence to medical treatment have been found to be less than desired [4-6]. It is influenced by the severity of the disease, the duration of the ailment, the number of eye drops being used, the literacy level, and the cost of medication [7-9]. To the best of our knowledge, a study of less than 100 Saudi glaucoma patients at a university hospital was carried out in 2012 [10]. In view of changes in glaucoma medications in the last 10 years, the client’s acceptance among Saudi glaucoma patients is likely to change as compared to the past and is worthy of study.

We present the adherence rate of topical medications and its determinants among adult Saudi glaucoma patients getting treated in 2017 in a tertiary eye center in Saudi Arabia.

## Materials and methods

This cross-sectional survey was carried out from January to March 2017 at a tertiary eye hospital in Riyadh, Saudi Arabia. The institutional research board of the university approved this study. This being a survey-based study, written consent was waived. However, informed oral consent was obtained from all participants. The study was undertaken following the strict norms of the Helsinki declaration for research. Adult glaucoma patients declining to participate were excluded from the survey, and they were assured about their high-quality glaucoma care despite their refusal. The participant’s personal identity was delinked from the result during the analysis.

To undertake a cross-sectional study, we assumed that among 1,300 patients visiting the eye department of a tertiary eye hospital, the level of non-adherence with glaucoma medication would be in 27% of glaucoma patients. To achieve a 95% confidence interval and an acceptable error margin of 5% for a survey, we needed to interview at least 253 participants.

Five medical students who were trained to undertake the interview of glaucoma patients were our field investigators. In our study, we defined non-adherence with glaucoma treatment based on client-perceived responses regarding questions on missing medication doses during the last two months. We also inquired judiciously about abiding with the appointments for follow-ups with glaucoma specialists.

The demographic information included age, gender, nationality, area of residence, education, status, and family history of glaucoma. Information about glaucoma-related barriers included the duration of glaucoma, past history of surgeries, and the number of glaucoma medications being used.

A pretested questionnaire in the Arabic language was used to enquire about adherence with glaucoma medication and possible risk factors for compliance. In view of the free cost of medication, as well as the ophthalmic services available in Saudi Arabia, we did not include cost as one of the barriers to adherence.

The data were transferred from forms to a Microsoft Excel spreadsheet (Microsoft Corporation, Redmond, Washington). Based on the day of the interview and the date of birth, age in years was calculated. The date of the last follow-up and the date of glaucoma diagnosis was used to estimate the duration of glaucoma treatment. It was further categorized as within one year, one to five years, and more than five years. We transferred the data in spreadsheet form to Statistical Package for Social Sciences (SPSS 23; IBM Corp., Armonk, NY). Frequency distribution and percentage proportion was the method of presentation of qualitative variables. For quantitative variables such as age, we plotted the distribution and if it was normal, we calculated the mean and standard deviation. In cases of uneven distribution, we presented the median and 25% quartile values. To compare the adherence rate among subgroups, we used 2 x 2 tables and estimated the odd’s ratio, 95% confidence interval, and two-sided p-value. If p was less than 0.05, we considered it statistically significant.

## Results

The profile of the participants is given in Table 1. Two-thirds were school graduates, Saudi nationals, and had undergone surgery for glaucoma in the past (e.g., YAG laser peripheral iridotomy (PI)). Most (57%) had glaucoma for more than one year.

**Table 1:** Profile of adult glaucoma patients being treated with glaucoma medications

Variables		Number	Percentage
Gender	Male; Female	152; 111	57.8; 42.2
Education	Read and write less than Bachelor; Bachelor; More than Bachelor	156; 66; 39; 2	59.3; 25.1; 14.8; 0.8
Nationality	Saudi; Non-Saudi	202; 61	76.8; 23.2
Resident	Riyadh; Outside Riyadh	177; 86	67.3; 32.7
Glaucoma diagnosed	Within 1 year; 1 to 5 years; More than 5 years	115; 96; 52	43.7; 36.5; 19.8
Glaucoma being treated	Yes; No	256; 7	97.3; 6.7
Number of glaucoma medications prescribed	1; 2; 3; More than 3	54; 141; 54; 14	20.5; 53.6; 20.5; 5.3
Glaucoma surgery in the past (including PI)	Yes; No	200; 63	76.0; 24.0
Glaucoma in relatives	Yes; No	141; 122	53.6; 46.4
Age (years)	Mean; SDV	61.6; 14.2	

The client-perceived subjective adherence rate to glaucoma medication was noted in 191/263 = 72.6% (95% confidence interval 67.2-78.0). Of the 263 glaucoma patients, 229 carefully attended follow-up appointments with ophthalmologists. Thus, the compliance to follow-up for glaucoma treatment was 87.1% (95% CI 83.0 - 91.1).

The demographic details and risk factors were associated with glaucoma medication adherence (Table 2). Glaucoma patients with a shorter duration of the disease had significantly worse compliance with medical treatment than those with a longer duration of glaucoma. Other factors were not significantly associated with adherence. The adherence rate in this present study was compared to other studies in the literature (Table 3).

**Table 2:** Adherence to the glaucoma medication among adult Saudi patients and their statistics

Determinants		Adherence (N = 191)		Non-adherence (N= 72)		Validation
		Number	Percentage	Number	Percentage	
Gender	Male; Female	107; 84	70.4; 75.7	45; 27	29.6; 24.3	OR = 0.76 (95% CI 0.4 – 1.3), p = 0.3
Education	School graduate; Higher education	109; 82	57.1; 42.9	47; 25	65.3; 34.7	OR = 0.7 (95% CI 0.4 – 1.2), p = 0.2
History of glaucoma surgery	Yes; No	146; 45	76.4; 23.6	54; 18	75; 25	OR = 1.1 (95% CI 0.6 – 2.0), p = 0.8
Residence	Riyadh; Outside Riyadh	129; 62	67.5; 32.5	48; 24	66.7; 33.3	OR = 1.0 (95% CI 0.6 – 1.9), p = 0.9
Using glaucoma medications	1 to 2; 3 and more	140; 51	73.3; 26.7	55; 17	76.4; 23.6	OR = 0.8 (95% CI 0.5 – 1.6), p = 0.6
Relative suffering from glaucoma	Yes; No	97; 94	50.8; 49.2	44; 28	61.1; 38.9	OR = 0.7 (95% CI 0.4 – 1.1), p = 0.1
Age	Mean SDV	61.3; 14.9		62.4; 12.2		p = 0.6
Duration of glaucoma	Within 1 year; 1 to 5 years; More than 5 years	69; 75; 47	36.1; 39.3; 24.6	46; 21 5	63.9; 29.2; 6.9	χ^2^ = 17.8, df*=3, P <0.001

**Table 3:** Adherence to glaucoma medication among adult Saudi glaucoma patients compared to other studies.

	Author	Year	Sample size	Adherence rate	Notes	Reference
1	Khandekar R	2005	105	25% compliance	Poor KAP as a cause	9
2	Osman EA	2016	108	81.6%	Duration of medication and barrier	10
3	Masouri K	2011	200	81%	Glaucoma patients in Switzerland	12
4	Mehri T	2016	359	42.6%	Cost, education level and barriers	13
5	Newman Casey	2015	1234	23% after 4 years	Duration, age and cost barriers	11
6	Cohen Costel O	2014	738	71%	Duration, age, and barriers	14
7	Boland MV	2014	407	82.8%	Objective method, 3 months after the start of medication	15
8	Lunnela J	2011	249	67%	Finnish glaucoma patients	16
9	Movahedinejad T	2016	130	34%	Education, rural patients barrier	17
10	Jiang H	2017	156	53.2	Complex medications barrier	18

## Discussion

Our study, which has a large sample, is perhaps one of the first studies to assess compliance with medical treatment among adult Saudi glaucoma patients. The adherence rate for topical glaucoma medication measured in the subjective method was 72% among adult glaucoma patients. Adherence with the follow-up appointment with the glaucoma specialist was as high as 87%. Those with less than a one-year duration of glaucoma treatment had a significantly poorer adherence rate with glaucoma medication usage as compared to patients with a longer duration of ailment.

Adult glaucoma patients that we studied are unique, as ophthalmic services and medications to Saudi residents is available free of cost or the cost is reimbursed by the insurance company in most cases. Thus, the cost of medication is less likely a barrier resulting in non-adherence with the medical treatment of glaucoma in this population.

Adherence with medical treatment found in the present study versus the literature suggested that despite different sets of barriers, the adherence rate in Saudi adult glaucoma patients noted in our study was high [10,11-18]. It was as low as 25% in Oman and as high as 81% in Switzerland. Different methods (subjective and objective methods of assessment and the variation of barriers in different countries could explain this wide variation in adherence rate). Identifying the barriers and addressing them could improve the adherence rate for taking prescribed glaucoma medications.

A shorter duration of glaucoma was significantly associated with the low adherence rate in our study. This was noted in a study where compliance was studied in the first and fourth years [11]. Cost implications and the human behavior of lethargy after some time could be the reason for poor adherence with glaucoma medications. In spite of having free access to medication and ophthalmic services, the non-adherence of 13% of glaucoma patients needs to be reviewed in further detail to address them.

The number of medications is a representation of the complexity of the glaucoma medication regimen. In our study, most cases were using one or two medications to manage their glaucoma. Newer, long-lasting medications that need to be instilled once a day, although costly, are free for Saudi patients, and this could explain the differential adherence found in our study and other studies [19-20].

Although the adherence rate of glaucoma medication was better in males as compared to females in our study, the element of chance observation cannot be ruled out. Gender was not a risk factor for adherence in China [18].

Age was not associated with the adherence rate in our study. However, researchers from Iran noted it as a significant risk factor [17]. Perhaps one of the barriers affecting older patients is cost, as seen in another study but not in Saudi Arabia. Having free-of-cost health services for older Saudi patients with a mean age of 62 as compared to glaucoma patients in Iran (mean 53) could explain age being a differential barrier for adherence.

A number of demographic and social factors have been associated with adherence to glaucoma medications [21]. In western countries, the elderly population is living in isolation and not having family support in contrast to Saudi society; therefore, the self-reliance of doses and instillation of eye drops could be an important difference, which we did not find significantly affecting the adherence rate in our study.

Knowledge, beliefs, and attitude is known to affect the adherence rate. In our study, the education level and a relative having glaucoma were not associated with the adherence rate. This indirectly suggests that knowledge about the use of medication in the treatment of glaucoma, which is gained by patients, had a limited influence on adherence in our glaucoma patients. Modes of dispersing knowledge to elderly glaucoma patients and their impact on adherence rates to medications for glaucoma management need to be further studied.

We had a few limitations to our study. Self-reporting on adherence was used as a method used similarly in many other studies. However, the objective method, a more reliable mode of measuring the adherence rate, has been tested for a short duration (three months) of glaucoma treatment [15,22]. It could be better to determine the negative effect of non-compliance on the progression of glaucoma. But the practical implications of using such a method in many cases where medications are to be used lifelong are questionable. There are a number of subcomponents of non-compliance to the medical treatment of chronic diseases [9]. We assessed only self-reported misses of doses and ophthalmic appointments. This could have resulted in unintentionally underestimating nonadherence.

The adherence rate in this present study was compared with other studies in the literature (Table 4).

**Table 4:** Adherence with glaucoma medication among adult Saudi glaucoma patients as compared to other studies

	Author	Year	Sample size	Adherence rate	Notes	Reference
1	Khandekar R	2005	105	25% compliance	Poor KAP as a cause	9
2	Osman EA	2016	108	81.6%	Duration of medication and barrier	10
3	Masouri K	2011	200	81%	Glaucoma patients in Switzerland	12
4	Mehri T	2016	359	42.6%	Cost, education level and barriers	13
5	Newman Casey	2015	1234	23% after 4 years	Duration, age, and cost barriers	11
6	Cohen Costel O	2014	738	71%	Duration, age, and barriers	14
7	Boland MV	2014	407	82.8%	Objective method, 3 months after the start of medication	15
8	Lunnela J	2011	249	67%	Finish glaucoma patients	16
9	Movahedinejad T	2016	130	34%	Education, rural patients barrier	17
10	Jiang H	2017	156	53.2	Complex medications barrier	18

## Conclusions

In conclusion, adherence is influenced by the severity of the disease, duration of the ailment, number of eye drops being used, literacy level, and cost of medication. The adherence rate for topical glaucoma medication was 72% among adult glaucoma patients, and the adherence to follow-up appointments with a glaucoma specialist was as high as 87%. The adherence rate in adult Saudi glaucoma patients was high, which is encouraging; however, a better assessment method than self-reporting is recommended to confirm the findings. The use of modern tools for better patient counseling and the addressing of identified barriers could make further inroads in delaying severe blinding complications of glaucoma thus affecting the quality of life. More work is needed to study knowledge distribution to elderly glaucoma patients and its impact on medication adherence rates.
